# Efficacy and In-Use Tolerance of Venusia Baby Moisturizer for Skin Hydration in Babies With Dry and/or Normal Skin

**DOI:** 10.7759/cureus.45032

**Published:** 2023-09-11

**Authors:** Neha, Rajiv Joshi, Amit Bhave, Seema Vikas Bhagat, Krishna Veligandla, Rahul Rathod, Bhavesh Kotak

**Affiliations:** 1 Medical Affairs, Dr. Reddy's Laboratories Ltd., Hyderabad, IND; 2 Dermatology, C.L.A.I.M.S Pvt. Ltd., Mumbai, IND; 3 Pediatrics, C.L.A.I.M.S Pvt. Ltd., Mumbai, IND

**Keywords:** parent perception, pama free, clinical evaluation, moisture meter sc, mean skin hydration, indian study on babies, dry skin

## Abstract

Introduction

Skin hydration is important for maintaining adequate skin barrier function. After delivery, the baby's skin faces the most difficult challenge as they are exposed to the exterior world's environmental changes, friction, and microorganisms. The management is further complicated by the availability of a large range of infant skin-care products with varying claims. The first-ever Indian study on babies was done to analyze the test product (Venusia baby moisturizer; Dr. Reddy's Laboratories Ltd., Hyderabad, India) in order to bring scientific clarity to consumers. This product is devoid of parabens, alcohol, and animal origin (Dr. Reddy's Laboratories Ltd., Hyderabad, India) and is designed for skin hydration and in-use tolerance in babies with dry and/or normal skin. The endpoints were hydration and clinical evaluation of the skin, evaluated using a moisture meter scale (MMSC; Delfin Technologies Ltd., Kuopio, Finland) and parent self-assessment questionnaire, respectively.

Material and methods

A total of 136 healthy babies aged between six months to two years were enrolled in a four-group, monocentric, non-randomized, evaluator-blinded study: Group 1 (Venusia baby cream for dry skin), Group 2 (Venusia baby lotion for Dry Skin), Group 3 (Venusia baby cream for normal skin), and Group 4 (Venusia baby lotion for normal skin). The endpoints were hydration and clinical evaluation of the skin, evaluated using an MMSC and parent self-assessment questionnaire, respectively.

Results

In babies with dry skin, skin hydration was improved with Venusia baby cream (37.50%) and Venusia baby lotion (66.40%). Additionally, 66.66% of participants strongly agreed that the baby’s skin became softer and smoother after the application of Venusia baby cream; 76.47% of participants strongly agreed that the baby’s skin became softer and smoother after the application of Venusia baby lotion. In babies with normal skin, skin hydration was improved with Venusia baby cream (12.20%) and Venusia baby lotion (7.20%); 59.37% of participants strongly agreed that the baby’s skin became softer and smoother after the application of Venusia baby cream; and 84.84% of participants strongly agreed that the baby’s skin became softer and smoother after the application of Venusia baby lotion.

Conclusion

Significant improvement was seen in skin hydration using Venusia baby cream and Venusia baby lotion in babies with dry skin and normal skin. No skin intolerances and product-related adverse or serious adverse events were clinically observed or reported during the study duration. Venusia baby lotion had the highest effect (66.4%) on skin hydration in babies with dry skin, where there was a significant shift from dry skin to normal skin range.

## Introduction

Human skin has an important role in maintaining internal moisture balance as it functions as a barrier, thereby preventing water loss. [1]. The stratum corneum (SC) (outer layer of the skin) functions properly when the skin is healthy [2,3]. The presence of optimum water content in the SC is essential for flexible, soft, smooth, and healthy skin [4]. In term infants, the skin barrier is not fully developed, and it undergoes development up to 12 months after birth [1]. Because of this developing state, infant skin is prone to dryness and more liable for damage from environmental factors, such as cold and windy climates, which in turn makes it more susceptible to skin barrier disruption [5].

Defects in the barrier nature of the epidermis, decreased levels of the lipids present between cells, and a reduction in natural moisturizing factors can lead to disruption of the barrier function of the epidermis, which can increase the likelihood of developing or exacerbating eczema. Therefore, repairing damaged skin barriers has become crucial in both preventing the development of eczema and in the management of eczema. Moisturizing leads to reduced itching, improves the skin's barrier function, minimizes the need for topical corticosteroids, and reduces the frequency of flare-ups during treatment for eczema [6].

More than 50% of newborns have skin problems, such as dry skin, atopic dermatitis (AD), contact dermatitis, diaper dermatitis, seborrheic dermatitis, psoriasis, and prickly heat. Eczema is the most commonly prevalent dermatitis; it is also known as AD [7]. Eczema or AD is a chronically relapsing condition characterized by a decrease in the hydration levels of the skin and suboptimal integrity of the skin [8]. In children, the lifetime prevalence of AD amounts to 15-30%. The majority of cases (60%) occur in the first year after birth. Psoriasis is a chronic autoimmune condition mostly attributed to genetic causation and further affected by environmental factors. Hydration of the skin and topical anti-inflammatory medication during flare-ups are the main modalities of treatment for this condition. The priority in treatment is the regular application of a moisturizer that has limited preservatives [7,8].

Moisturizers increase hydration, leading to enhanced water content in the SC; this, in turn, leads to skin that feels and looks smoother. Moisturizers can be classified based on their water content into lotions (high water content) or creams (less water content and more oils or occlusive agents and perform very important functions in babies. The main function of topical moisturizers is to form a protective layer on the surface of the skin; this prevents water loss from subcutaneous tissue and increases hydration. Other functions of topical moisturizers are the maintenance of subcutaneous tissue integrity and the prevention of hypothermia by enhancing barrier function [5]. Regardless of the baby’s skin type, it is important to choose a moisturizer that is gentle, hypoallergenic, and free from fragrances and harsh chemicals.

There is still a lack of clarity and gaps in knowledge in understanding baby skin disorders (AD, psoriasis). Apart from lotions and creams as mentioned above, the other formulations include ointments, gels, oils, and sprays. Physicians should educate parents regarding the importance and necessity of moisturizer usage in the management and prevention of eczema. Physicians need to emphasize the role of moisturizers in lessening eczema flare-ups, reducing the need for topical corticosteroids, shortening treatment duration, and decreasing the frequency of flare-ups [6]. Skin hygiene with a liberal application of emollients is an important topic of education for parents. Currently, the clinical significance of moisturizers is appropriately documented furthermore positioning plant-based natural moisturizers as a preferable first-line treatment [9,10].

Currently, there are a wide number of baby moisturizing creams available in the Indian market with no proven safety profile. In order to provide scientific and clinical clarity this first-ever Indian study on babies was done to examine the status of skin hydration and in-use tolerance of Venusia baby moisturizer.

## Materials and methods

Study design and setting

This was a four-group, monocentric, non-randomized, evaluator-blinded study. A total of 136 participants were divided into four groups: Group 1 (Venusia baby cream for dry skin), Group 2 (Venusia baby lotion for dry skin), Group 3 (Venusia baby cream for normal skin), and Group 4 (Venusia baby lotion for normal skin). Venusia baby cream and lotion is manufactured by Delfin Technologies Ltd. (Head Office: Kuopio, Finland) and is a plant-based moisturizer that contains a unique combination of four butters: shea, cocoa, mango, and aloe. Informed consent was obtained from the parents or caregivers of the participant before performing any study-related procedure. The study was approved by the Institutional Ethics Committee and conducted respecting the principle of the Declaration of Helsinki and its amendments in conformity with the Good Clinical Practices and New Drugs and Clinical Trials Rules, 2019. Healthy babies aged between six months to two years (inclusive) with either normal skin (moisture meter scale (MMSC; Delfin Technologies Ltd., Kuopio, Finland) reading >20%) or dry skin (MMSC readings <20%) were included in the study. The exclusion criteria included babies having a chronic illness or any clinically significant systemic/cutaneous disease (presently or in the past one month), using any moisturizing products on test sites (legs) from three days before the study, history of allergic dermatitis or contact allergy to cosmetics, and hypersensitivity to any cosmetic products or raw materials.

Study plan and outcomes

The study was conducted to evaluate skin hydration after the application of test products - Venusia baby cream (paraben-free; Batch number: BZD116) and Venusia baby lotion (paraben-free; Batch number: BZD2027; Dr. Reddy's Laboratories Ltd, Hyderabad, India) - on the entire body. During clinical evaluation, the test site (legs) was cleaned with soap and water, and the babies were acclimatized under controlled conditions for 30 mins. The humidity was maintained between 40% and 60%, and the temperature at 20-22 °C. MMSC measurements were carried out on the test sites, followed by a clinical examination of the skin.

The study outcomes were skin hydration of SC measured using MMSC and clinical evaluation by a dermatologist for the overall in-use skin tolerance of the test product done by using the Draize scale. Safety outcome was evaluated through clinical examination of the skin for any adverse reaction after test product application.

Through the parent questionnaire, an evaluation of the moisturizer was done based on parameters such as product spreadability and absorption, non-sticky feel, softness and smoothness of skin post-application, no redness after application, and overall satisfaction/acceptance.

Statistical methods

Statistical analysis was carried out by using the 10.0 version of Statistical Product and Service Solutions (SPSS) software (IBM SPSS Statistics for Windows, Armonk, NY). Continuous variables were summarized by treatment group using summary statistics (number of observations, mean, standard deviation, or median with range of minimum and maximum). Tests of significance used were the Student's t-test. Qualitative data were captured in tables with representative figures. All p-values were reported and interpreted at a 5% level of significance.

## Results

A total of 136 participants were included in the study. Participants were divided into four groups. The mean (SD) age of the recruited participants was 16.03 (05.63) months (range: 06-23 months). Out of 136, 132 participants completed the study with four dropouts. Data were analyzed for 132 participants. Skin hydration was measured using MMSC reading at the initial and end of the study (two weeks).

Out of 132 participants, 65 participants used Venusia baby cream, and 67 participants used Venusia baby lotion. A total of 32 babies with normal skin and 33 babies with dry skin used Venusia baby cream, and 33 babies with normal skin and 34 babies with dry skin used Venusia naby lotion (Table 1).

**Table 1:** Demographic data - Venusia baby cream and lotion for normal and dry skin (Groups 1, 2, 3, and 4)

Parameters	Group 1 (No. of cases)	Group 2 (No. of cases)	Group 3 (No. of cases)	Group 4 (No. of cases)
n	33	34	32	33
Mean	16.03	14.91	14.63	16.18
SD	05.63	05.13	06.00	04.67
Age range (months)	06.00-23.00 months	06.00-23.00 months	06.00-23.00 months	07.00-23.00 months
Gender (%)				
Male	17 (51.5%)	17 (50.0%)	17 (53.1%)	18 (54.5%)
Female	16 (48.5%)	17 (50.0%)	15 (46.9%)	15 (45.5%)

After the application of Venusia baby cream on dry skin participants for two weeks, mean skin hydration showed a significant increase of 37.5% from baseline (Table 2).

**Table 2:** Changes in mean skin hydration in normal and dry skin participants using Venusia baby lotion/cream (Groups 1, 2, 3, and 4) By Student's t-test: *Significant

Parameters	Group 1 (No. of cases)	Group 2 (No. of cases)	Group 3 (No. of cases)	Group 4 (No. of cases)
Duration in weeks	Mean skin hydration (X±SD) (N=33)	Mean skin hydration (X±SD) (N=34)	Mean skin hydration (X±SD) (N=32)	Mean skin hydration (X±SD) (N=33)
Baseline	12.55 ± 03.44	12.59 ± 03.46	28.39 ± 05.78	29.37 ± 06.91
2 Weeks	17.26 ± 04.75	20.95 ± 07.73	31.87 ± 06.82	31.47 ± 07.38
Mean diff (baseline-2 weeks)	*04.71 ± 04.07	*08.36 ± 06.88	*03.47 ± 05.07	*02.10 ± 03.05
P-value	0.001	0.001	0.001	0.001

Venusia baby cream for dry skin (Group 1)

In Group 1, there were 33 participants. The age of the participants ranged from 06.00 to 23.00 months with a mean SD age of 16.03 (05.63) months. Of 33 participants, 17 (51.5%) were boys, and 16 (48.5%) were girls (Table 1).

The mean (SD) values of skin hydration at baseline and after two weeks were 12.55 (03.44) and 17.26 (04.75), respectively. After the application of Venusia Max Cream on dry skin participants for two weeks, mean skin hydration showed a significant increase of 37.5% from baseline (Table 2).

Venusia baby lotion for dry skin (Group 2)

In Group 2, there were 34 participants. The ages of the study participants ranged from 06.00 to 23.00 months, with a mean SD age of 14.91 (05.13) months. Of 34 participants, 17 (50%) were boys, and 17 (50%) were girls (Table 1).

The mean (SD) values of skin hydration at baseline and after two weeks were 12.59 (03.46) and 20.95 (07.73), respectively. After the application of Venusia baby Lotion on dry skin participants for two weeks, mean skin hydration showed a significant rise of 66.4% from baseline (Table 2).

Venusia baby cream for normal skin (Group 3)

In Group 3, there were 32 participants. The ages of the study participants ranged from 06.00 to 23.00 months, with a mean SD age of 14.63 (06.00) months. Of 32 participants, 17 (53.1%) were boys, and 15 (46.9%) were girls (Table 1).

The mean (SD) values of skin hydration at baseline and after two weeks were 28.39 (05.78) and 31.87 (06.82), respectively. After the application of Venusia baby cream on normal skin participants for two weeks, mean skin hydration showed a significant rise of 12.2% from baseline (Table 2).

Venusia baby lotion for normal skin (Group 4) 

In Group 4, there were 33 participants. The ages of the study participants ranged from 07.00 to 23.00 months, with a mean SD age of 16.18 (04.67) months. Of 33 participants, 18 (54.5%) were boys, and 15 (45.5 %) were girls (Table 1).

The mean (SD) values of skin hydration at baseline and after two weeks were 29.37 (06.91) and 31.47 (07.38), respectively. Based on the statistical interpretation, Venusia baby lotion on normal skin participants after the application of two weeks showed a significant rise in mean skin hydration of 7.2% from baseline (Table 2).

**Figure 1:**
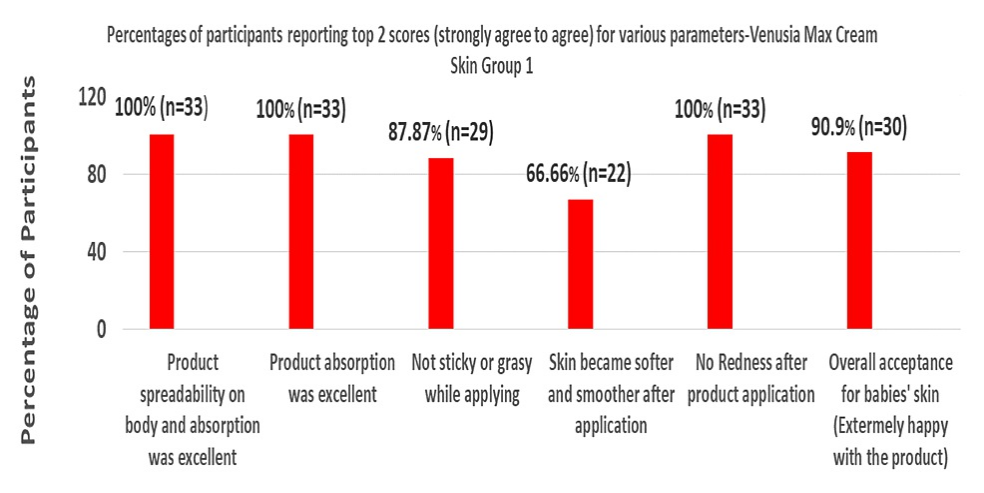
Percentages of participants reporting top 2 scores (strongly agree to agree) for various parameters for Venusia baby cream for dry skin (Group 1)

**Figure 2:**
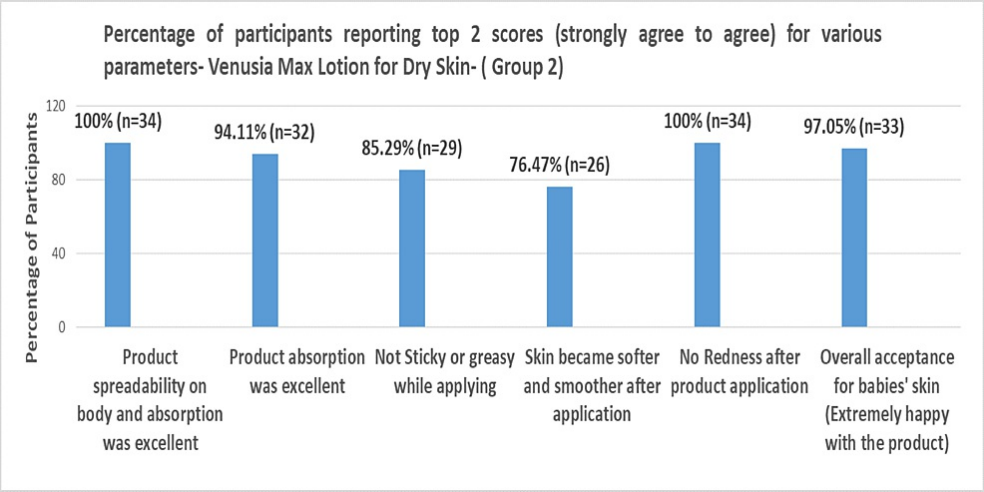
Percentages of participants reporting top 2 scores (strongly agree to agree) for various parameters for Venusia baby lotion for dry skin (Group 2)

**Figure 3:**
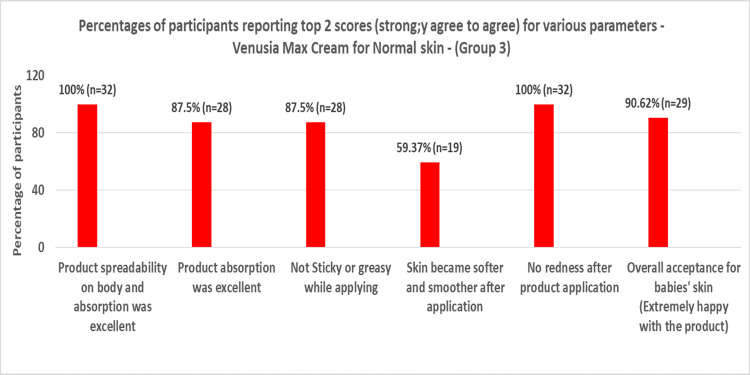
Percentages of participants reporting top 2 scores (strongly agree to agree) for various parameters for Venusia baby cream for normal skin (Group 3)

**Figure 4:**
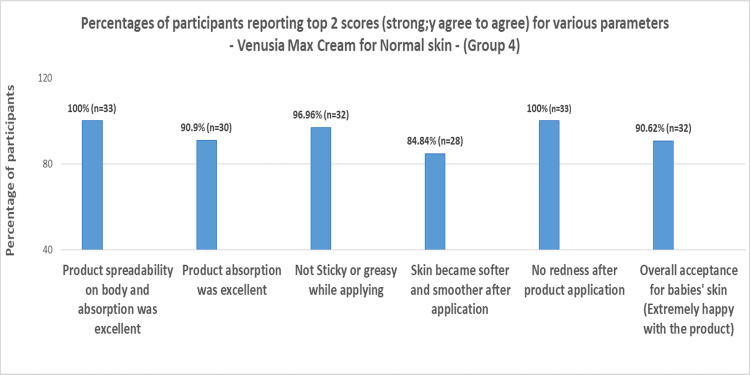
Percentages of participants reporting top 2 scores (strongly agree to agree) for various parameters for Venusia baby lotion for normal skin (Group 4)

Based on the parent questionnaire evaluation, the percentage of participants reporting top 2 scores (strongly agree to agree) in Groups 1, 2, 3, and 4 can be summarized in Figures 1-4 below, respectively.

Safety/adverse events

There were no serious adverse events reported during the entire study duration. None of the trial subjects reported any signs of redness or rash.

## Discussion

This is the first study of its kind to be conducted in India on the effectiveness and tolerability of moisturizer when applied to infant skin while measuring skin hydration and in-use tolerance.

For babies, moisturizer is a crucial part of fundamental daily skin care. Through a direct supply of water from the skin's water phase, moisturizers enhance skin hydration and raise the water content in the SC. It also improves occlusion to stop trans-epidermal water loss, protecting skin integrity. Utilizing a moisturizer also makes the skin's surface smoother, thereby resolving dry skin issues [11,12]. Therefore, regardless of the existence of dermatitis and/or dry skin, moisturizers should be given regularly and consistently to children with eczema, with application to the entire body [6].

Variable amounts of humectants (such as urea or glycerol), physiological lipids, and occlusives are used to make moisturizers [13]. Glycerine-stimulated enzymes reduce itching, while dimethicone (emollient) softens and moisturizes skin and lessens itchiness and flaking while offering a barrier of defense against irritants. Shea butter is another ingredient shown to benefit patients with AD. Moisturizers with a blend of glycerine and cocoa butter are found effective in soothing inflamed skin. Additionally, mango butter has been shown to potentially reduce skin irritability [14]. Venusia baby moisturizer with a unique combination of shea, cocoa, mango, and aloe butter in the formulation of a single lotion/cream makes an effective moisturizer with anti-inflammatory properties that are beneficial and effective in patients with AD.

The goal of the current study was to assess the skin hydration of infants following the application of a test product (Venusia baby moisturizer, a plant-based natural moisturizer free of paraben, alcohol, phthalates, and animal origin), as well as the product's tolerability during use. Parents or caregivers were provided with a weighed amount of moisturizer to be applied liberally over the body two to three times a day. During the trial, evaluations were conducted utilizing the MMSC instrument to assess skin hydration. A clinical examination was done to determine whether there were any skin intolerances, and a questionnaire was used to record parents' perceptions of the product.

Our research revealed that, when Venusia baby cream and Venusia baby lotion were applied directly to dry skin, significant improvements in skin hydration were seen in 37.50% and 66.40%, respectively. The effectiveness of Venusia baby cream and Venusia baby lotion for normal skin was 12.20% and 7.20%, respectively. These results were in contrast to a study by Chiang et al. that assessed the skin's level of moisture following various combinations of bathing and moisturizing routines in children with AD and with healthy skin [15]. Using Standard capacitance measurements, Chiang et al. showed a moderate increase in hydration by applying moisturizer after bathing; however, this increase was proven to be less than that attained by doing so alone [15].

The extent of safety and tolerability of a skin-restoring body wash and skin-restoring moisturizer for infants and toddlers with AD was examined in another study by Simpson et al., where 72.7% of parents concurred that the application made the baby's skin softer and smoother [16]. In contrast, the current study with Venusia baby moisturizer indicated that 76.47% of participants strongly agreed that the baby's skin improved after the application of Venusia baby lotion for dry skin. Additionally, the noticeable increase in skin hydration observed demonstrated that Venusia baby moisturizer outperformed Restoraderm.

At any time during our trial, none of the patients reported any negative events, erythema, or edema with Venusia baby lotion/cream. In contrast, the study by Simpson et al. found that using their skin-replenishing moisturizer caused unpleasant reactions such as body rash, erythema, noticeable scaling on the face, exacerbation of AD, and impetigo [16].

When Venusia baby cream and baby lotion were applied to infants or toddlers with dry skin or normal skin, the product had exceptional spreadability over the body, and 100% of participants strongly affirmed that there was no redness. More than 85% of participants in Groups 1 and 2, 87.5% of participants in Groups 3 and 4, and more than 95% of participants in Group 4 firmly agreed that the product was not oily. The majority of participants (66.66% in Group 1, 76.47% in Group 2, 57.37% in Group 3, and 84.84% in Group 4) agreed that the baby's skin grew softer and smoother following application. In all groups, about 90% of participants definitely stated that the product showed great absorption.

Our results were superior to a previously conducted single-center, open-label trial by Simpson et al. (2012), which evaluated the safety and tolerability of a skin-restoring body wash and moisturizer in infants and toddlers with AD. Only 72.7% of parents of participants in the study by Simpson et al. agreed that the baby's skin becomes supple and smooth [16].

Treatment of AD and eczema in children involves educating the parents, as well as the caregivers, because of the chronicity of the condition, as well as the requirement of ongoing management. An earlier study found that parental understanding of moisturizers affects the use of these products in neonates with eczema. Almost 72% of parents were aware that moisturizers could help children with eczema rebuild the skin barrier, and 50% of parents were aware that moisturizers can assist in restoring the skin barrier that has been damaged. Only 7.4% of parents were aware that moisturizers might lessen skin sensitivity, while 8.6% were aware that they could lessen persistent repeated episodes. These findings show that many parents lacked an awareness of the role of moisturizers in the management and prevention of eczema, which may cause them to overlook the importance of moisturizers [6].

In a study on parental knowledge of moisturizers, Li et al. [6] found that 57.9% (62/107) of parents did not use moisturizers, 76.3% (161/212) did not use them regularly, and 75.8% (172/227) did not use them on their entire bodies because they were worried about the potential side effects. Parents stopped regularly applying moisturizers as a result of this unjustified worry [6].

These studies emphasize that parent education is essential for proper skin care and avoidance of triggers for long-term management and care of children with these skin conditions. Although parent education was not an active component of our study, we propose it as an integral part of the treatment plan to help alleviate symptoms and prevent flare-ups.

Pediatricians can play a significant part in educating people about the proper use of moisturizers in this situation. According to British research, teaching parents how to properly apply moisturizers increased the number of moisturizers used by 800%, reduced the severity of eczema, and decreased the proportion of patients who needed moderate or powerful topical steroids. To guarantee proper adherence from parents, pediatricians should play a significant role in educating parents on the frequency of application, dose, formulation, and adverse effects of moisturizers [6]. Patients' first option for AD prevention and treatment should be daily application of full-body emollient therapy [17].

The preventative effect of moisturizer is greatest during the first month of latency, with the remaining 47% risk reduction occurring by the third month, according to Kritsanaviparkporn et al. [12]. Moisturizers can be used to both prevent and treat AD, according to experts. Studies have shown that using moisturizers often during the neonatal stage, especially in high-risk infants, lowers the risk of developing AD later in life [15].

Daily use of moisturizers reduces the relative risk of AD incidence by up to 50%, according to Michael et al. [17]. Horimukai et al. presented similar findings by conducting a prospective, randomized controlled study with daily moisturizer application to 59 of 118 newborns at high risk of AD during the first 32 weeks of life. This study showed a statistically significant reduction in AD incidence during infancy [18].

Limitations

There were a few limitations to the study. First, the duration of the study was short for two weeks to observe long-term effects. Second, it can be difficult to determine the true impact of the moisturizer with a limited population. However, the study provided insights into the practicality and acceptability of using the moisturizer in real-life settings, which definitely adds value.

## Conclusions

Based on the aforementioned research, it was determined that both infants and toddlers with dry or normal skin saw a considerable increase in skin hydration when using Venusia baby cream and Venusia baby lotion, manufactured by Dr. Reddy's Laboratories Ltd., Hyderabad, India. The study was carried out by maintaining the humidity between 40% and 60% and temperature at 20-22 °C for the entire study duration. Throughout the course of the trial, there were no clinically seen or reported skin intolerances or serious adverse events associated with the product. In babies with dry skin, Venusia baby lotion had the greatest impact (66.4%) on skin hydration. The transition from dry skin to normal skin range represented a considerable change.

As Venusia baby moisturizer is plant-based and PAMA-free, it stands apart from most of the commercially available baby moisturizers with synthetic excipients. Further, based upon the results from this study, it can be concluded that, because of the demonstrated safety and efficacy, Venusia baby moisturizer is a promising natural plant-based agent for daily moisturization, as well as for dry skin conditions such as AD and psoriasis in babies.
